# Birth-Rates and World Wars

**Published:** 1921-02-05

**Authors:** 


THE PUBLIC WELL-BEING—(*continued*).

### Birth-Rates and World Wars.

Speaking recently at the annual meeting of the Mississippi Valley Medical Association, Dr. C. A. L. Reed, a well-known Cincinnati physician, made some remarkable statements with regard to the relation of birth-rates and world wars, which would have received the hearty approbation of that considerable body of expert opinion in England which strongly favours the adoption of a recognised form of birth control. Dr. Reed took the view, as the foundation of his remarks, that the underlying cause of the world war was the necessity for land and food by overpopulated countries. He describes the Treaty of Versailles as a "fatuous attempt to repeal the law of nature underlying racial existence"; and declared that if, instead of stimulating reproduction in violation of the fundamental laws of morality, the afflicted nations of Europe had formulated instructions to their peoples how voluntarily and safely to regulate their birth-rates and thus reduce their populations to a self-supporting basis and keep them there, if the Versailles plenipotentiaries had done this they would have given the world the only possible guarantee against war, and would have immortalised themselves by an act of transcendent statesmanship.

Dr. Reed's dismal view of the actualities is that the influences left uncorrected at Versailles make another world war inevitable, league or no league. It will be so much larger and more murderous than the last one, as the populations fighting for a place "in the sun" will be larger than those engaged in the last conflict, and it will come with a rapidity precisely relevant to the excess of birth-rates over death-rates. The birth-rate of the United States has been practically uninfluenced by the war. It is 22.3 per thousand of the population. This represents a decline of less than one-third of one point, and even this small decline is largely accounted for by the returns from the State of New York, in which alone there was a sharp fall from 24.2 in 1914 to 20.8 in 1919. The decline in New York is accounted for by the suspension of immigration, war service, and influenza, all of which conditions were especially acute in that State. If, therefore, one discounted the statistical aggregate for the whole country by these facts from New York, it is reasonable to assume that the national birth-rate in the States is rather higher since than before the war.

The following are Dr. Reed's conclusions: (1) The rapid growth of population everywhere, more rapid than is conducive to world welfare, justifies the teaching of safe contraceptive methods, and this teaching is a natural function as it is a moral obligation of the medical profession. (2) Contraceptive methods that are not hazardous either to the individual or to society are not only justifiable, but are demanded as safeguards against abortion and feticide, both of which ought to be punished by extreme penalty of the law. (3) Any statute that may interfere with the medical profession in the discharge of its duty in this or any other regard ought to be observed while it is on the statute books, but every possible effort should be made, not by the medical profession alone, but by society at large, to secure either the necessary amendment or enlightened judicial interpretation, or, in the absence of these, the repeal of such statute. (4) In the instance of persons applying for relief from infertility it is the professional duty of medical men, first, to disregard any sociologic views that they may entertain as applicable to the individual case; second, carefully to examine the case with reference to the active present cause; third, explain the situation frankly to the parties concerned; and fourth, bring all the resources of the medical and surgical art to bear upon a restoration of the function.

[Certainly the case for birth control, both from the point of view of the public and from that of the medical profession, has never been more clearly or fairly put.]

